# Strengthening scaling up through learning from implementation: comparing experiences from Afghanistan, Bangladesh and Uganda

**DOI:** 10.1186/s12961-017-0270-0

**Published:** 2017-12-28

**Authors:** Sara Bennett, Shehrin Shaila Mahmood, Anbrasi Edward, Moses Tetui, Elizabeth Ekirapa-Kiracho

**Affiliations:** 1 Department of International Health, Johns Hopkins Bloomberg School of Public Health, 615 N Wolfe St, Baltimore, MD 21205 United States of America; 2 ICDDR,B, Dhaka, Bangladesh; 3 Department of Health Policy Planning and Management, Makerere University School of Public Health, Kampala, Uganda; 4 Epidemiology and Global Health Unit, Department of Public Health and Clinical Medicine, Umeå University, 901 87 Umeå, Sweden

**Keywords:** Scaling up, Implementation research, Stakeholders

## Abstract

**Background:**

Many effective innovations and interventions are never effectively scaled up. Implementation research (IR) has the promise of supporting scale-up through enabling rapid learning about the intervention and its fit with the context in which it is implemented. We integrate conceptual frameworks addressing different dimensions of scaling up (specifically, the attributes of the service or innovation being scaled, the actors involved, the context, and the scale-up strategy) and questions commonly addressed by IR (concerning acceptability, appropriateness, adoption, feasibility, fidelity to original design, implementation costs, coverage and sustainability) to explore how IR can support scale-up.

**Methods:**

We draw upon three IR studies conducted by Future Health Systems (FHS) in Afghanistan, Bangladesh and Uganda. We reviewed project documents from the period 2011–2016 to identify information related to the dimensions of scaling up. Further, for each country, we developed rich descriptions of how the research teams approached scaling up, and how IR contributed to scale-up. The rich descriptions were checked by FHS research teams. We identified common patterns and differences across the three cases.

**Results:**

The three cases planned quite different innovations/interventions and had very different types of scale-up strategies. In all three cases, the research teams had extensive prior experience within the study communities, and little explicit attention was paid to contextual factors. All three cases involved complex interactions between the research teams and other stakeholders, among stakeholders, and between stakeholders and the intervention. The IR planned by the research teams focussed primarily on feasibility and effectiveness, but in practice, the research teams also had critical insights into other factors such as sustainability, acceptability, cost-effectiveness and appropriateness. Stakeholder analyses and other project management tools further complemented IR.

**Conclusions:**

IR can provide significant insights into how best to scale-up a particular intervention. To take advantage of insights from IR, scale-up strategies require flexibility and IR must also be sufficiently flexible to respond to new emerging questions. While commonly used conceptual frameworks for scale-up clearly delineate actors, such as implementers, target communities and the support team, in our experience, IR blurred the links between these groups.

**Electronic supplementary material:**

The online version of this article (doi:10.1186/s12961-017-0270-0) contains supplementary material, which is available to authorized users.

## Background

While the global health community frequently knows which interventions are effective, it is typically much harder to design policies and programmes that support the implementation of such interventions at scale [1]. Barriers to scaling up interventions exist at multiple levels of the health system, and may encompass factors such as lack of political support for the intervention, limited capacity to implement the intervention and inadequate demand from users [2]. The gap between interventions known to be effective and those which actually reach people in need is sometimes referred to as the ‘delivery gap’, and it has been argued that implementation research is the key to closing this gap [3]. This paper seeks to investigate how, in practice, implementation research can contribute to scaling up.

There has been a long-standing interest, both within and outside of the health sector, in how innovations get adopted and spread [4, 5]. Within the field of global health, interest in strategies for scaling up effective interventions has multiplied during the past decade as stakeholders have recognised the challenges in the scaling up of interventions to achieve the Millennium Development Goals [6]. Multiple frameworks have been developed to help analyse scaling up processes [2, 6–10], encompassing different but frequently overlapping dimensions of the scaling up process. For example, Hanson et al. [7] consider two primary dimensions, namely the level of the health system at which the constraint operates and the extent to which the constraint can be relaxed through the provision of additional funds. Subramanian et al. [6] identified five key dimensions of scaling up, namely the meaning of scaling up, the resources required to scale up, planning for scale-up, implementation perspectives, and monitoring and evaluation. Spicer et al. [10] adopted a more process-oriented approach, considering key activities for scale-up, including designing scalable innovations, planning scale-up, persuading governments to adopt innovations, supporting government to implement innovations, and promoting community acceptance of innovations.

The framework employed herein to consider critical elements of scaling up draws primarily upon two similar frameworks developed by Yamey [8] and Simmons et al. [9]. Specifically, we consider (1) the attributes of the innovation or service being scaled up, namely factors such as the simplicity versus complexity of the innovation and the scientific evidence base supporting the innovation; (2) the actors involved in scaling-up, including the target community, the implementers of the intervention, and the resource team that support on-the-ground implementers (possible considerations include the strength of local leadership and governance, the extent of local involvement and the diversity of actors involved); (3) the context, encompassing socio-political factors such as the extent of political support, country ownership and supportive national policies for the intervention or innovation, as well as the extent to which the environment supports learning; and (4) the delivery or scaling up strategy, concerning factors such as the use of phased approaches to scale-up and how the intervention is adapted to different localities.

Simmons et al. [9] suggested four primary scaling-up strategies, namely (1) vertical scaling up, which involves institutionalisation through policy, political, legal, budgetary or other health systems change; (2) horizontal scaling up, which implies expansion or replication of the programme or intervention in different geographic locations or healthcare facilities; (3) diversification, whereby new innovations are added to an existing intervention package, and the range of interventions provided are adapted; and (4) spontaneous scaling up, whereby diffuse actors adopt the intervention without any concerted plan or strategy. These strategies are not mutually exclusive and different types of strategy, especially vertical and horizontal scaling up, are likely to be required for sustainable scale-up.

While the topic of scale-up is of significant interest in its own right, there is also a strong association between approaches to scaling up and how sustainable an intervention may be over the long term. For example, ‘standalone’ strategies for scaling up, such as vertically organised programmes, can be effective in reaching large numbers of people quickly, but often fail to build the necessary health system capacities to sustain that service over time [11].

Several papers have called for greater investment in implementation research as a means to learn about effective strategies for scaling up health interventions and innovations in low- and middle-income countries [3, 12, 13]. As articulated by Madon et al. [13], if implementation is a viewed as an “*adaptive, dynamic, multiscale phenomenon*” then implementation research has a natural role to play in examining these processes. Indeed, going back as far as 1980, Korten [14] made a strong argument for the inappropriateness of blueprint approaches to scaling-up and instead argued that the key was not preplanning, “*but an organization with the capacity for embracing error, learning with the people and building new knowledge and institutional capacity through action*.” (p. 480).

Implementation research has been defined as “*the scientific inquiry into questions concerning implementation—the act of carrying an intention into effect, which in health research can be policies, programs, or individual practices (collectively called interventions)*” [15], and can ask a variety of questions about the policy or programme under investigation. While it is often relevant to assess the effectiveness of a policy or programme, implementation research may also seek to investigate its acceptability, appropriateness, adoption, feasibility, fidelity to original design, implementation costs, coverage and sustainability [15].

In this paper, we present case studies of how three Future Health Systems (FHS) research teams, in Afghanistan, Bangladesh and Uganda, used implementation research to address questions regarding the scalability and sustainability of the innovations that they were supporting. Specifically, we (1) analyse how implementation research contributed to understanding of the attributes of the service or innovation being scaled up, the scaling up strategy, the actors involved and the context, and thus informed the overall process of scaling-up and (2) reflect on the characteristics of implementation research strategies that make them best suited to support scale-up and sustainability, and how this may vary for different types of scaling up strategies.

At the time of writing, only one out of the three interventions described (that in Afghanistan) had been formally adopted as government policy and was in the process of being rolled out across the country. Our analysis therefore, focuses on how the implementation research informed intervention design and scale-up strategies, rather than the ultimate issue of whether scale-up was finally successful.

## Methods

FHS engaged in implementation research across five countries – Afghanistan, Bangladesh, China, India (specifically, the Sundarbans region in West Bengal) and Uganda – with the overarching goal of promoting more accessible, high-quality health services for the poor. This paper draws upon FHS experiences in Afghanistan, Bangladesh and Uganda. The second phase of FHS, which informs this analysis, took place in Bangladesh and Uganda in 2011–2016, and in Afghanistan in 2011–2014. This paper does not include the experiences of the FHS teams in India or China as in neither case was the team actively supporting the scale-up of an intervention. Rather, the FHS India team aimed to provide information about health conditions and service in the Sundarbans so as to stimulate local stakeholders to develop interventions and, in China, the FHS team was investigating how government health reforms were being implemented.

Our analysis sought to analyse the FHS-supported interventions across the four different dimensions of scale-up discussed above (Table 1). For each of these dimensions, we sought to understand how implementation research informed the scaling-up process, paying particular attention to the kinds of questions typically addressed by implementation research such as appropriateness and effectiveness, fidelity, acceptability, etc.

**Table 1:** Dimensions of scale-up and possible implementation research questions

Scaling-up dimensions informed by implementation research	Possible implementation research questions related to scaling-up
Attributes of the innovation or service	*Appropriateness:* How appropriate and effective is the intervention? How well does it fit with the context and actors? *Fidelity:* Did the attributes of the innovation remain the same as planned throughout the implementation process? How were they adapted over time?
Attributes of the target community and actors involved in scale-up	*Acceptability:* How acceptable is the intervention to key stakeholders? *Intention to adopt:* What are stakeholders intentions to adopt the intervention, and why is this the case? *Coverage:* Who is covered by the intervention? What are the attributes of those who are covered, and those who are not?
Context	*Feasibility:* Is it possible to implement the intervention in this context, given the resources available, the health systems structures, etc.?
Scaling-up strategy	*Implementation cost*: How much does it cost to deliver the intervention? Is this affordable? Could adaptations to design make the intervention more cost-effective? *Sustainability:* Is it possible to sustain the intervention once scaled up? What factors (intervention attributes, actors involved, context) contribute to or undermine sustainability?

Source: Adapted from Peters et al. [15]

For the three study contexts, we collected and collated information on the attributes of the innovation, the attributes of the target community and other actors involved (including the research team supporting the intervention), the context and the scaling-up strategy. Further, we sought to document how these factors were assessed or designed at the start of the project and how they evolved over the life of the project. Initially, we drew primarily upon project documentation to accomplish this. The lead author (SB) reviewed country team research proposals, country theories of change, completed both at the start of the project and then 3 years later (2014), country presentations at annual meetings, routine monitoring reports initially submitted monthly and then for the last 18 months of the project on a quarterly basis, annual reports submitted by the teams to the consortium management, and country publications, including articles and policy briefs, as the sources of project information.

Based upon these documentary sources we developed rich descriptions of the intervention and scale-up plans in each country addressing the different dimensions of the framework presented in Table 1 (Additional file 1). In addition, we extracted information on the nature of the implementation research conducted, what lessons had emerged from the implementation research and how this had affected the intervention and scale-up strategy. These rich descriptions were provided to each of the country study teams for their review and comment. Teams corrected misunderstandings or cast additional light on the choices they had made through the implementation and research process. In some cases, this led to further discussions in order to ensure an accurate representation of what had transpired. This paper synthesises findings across the three study countries, identifying common patterns and differences.

## Results

Tables 2, 3 and 4 present high-level summaries of the intervention, scale-up plans, implementation research and findings in each country. More information about the Afghanistan case can be found in Edward et al. [16], whereas information about the Bangladesh case in Khatun et al. [17], Khan et al. [18] and Khatun et al. [19], and on the FHS website [20]. It should be noted that the interventions in all three countries built upon a history of prior engagement. In Uganda, the research team has previously experimented with transport vouchers for pregnant women [21], in Bangladesh, there had been a history of engagement of Village Doctors (VDs) [22], and in Afghanistan, the team had worked with the Ministry of Public Health in developing a balanced scorecard reflecting the quality of health services [23].

**Table 2:** Afghanistan: interventions, scale-up strategy and implementation research

Scaling-up dimensions informed by IR	Nature of intervention and scale-up strategy	Findings from IR
Implementing team	FHS team (Johns Hopkins with local hires), in collaboration with three local NGOs and the community-based healthcare department of the MOPH	
Research aim and design	Aim to explore feasibility and effectiveness of community scorecards in Afghan context Three rounds of structured community and facility engagement processes involving identification and measurement of quality and performance indicators, and collective development of work plans; quarterly longitudinal data on indicators and qualitative evaluation using focus group discussions; findings on other dimensions emerged organically	
Attributes of the innovation or service	*Community level balanced scorecard* Aims: to enhance community engagement, accountability & responsiveness to users at the local level so as to improve quality of primary care services	*Appropriateness:* It was found easier for MOPH and NGO stakeholders to understand and appreciate the intervention if framed as an extension of the balanced scorecard, implemented in hospitals and health facilities; the Partnership Defined Quality strategy, similar to the Community Score Card, was also being implemented in Afghanistan, though community members were not involved in generating indicators or scores and therefore the scorecard was more readily accepted by the NGO teams *Fidelity:* Not assessed
Attributes of the target community	Initially implemented in Takhar and Bamyan provinces with ethnic Hazara, Uzbek and Tajik communities. Later (post-FHS) expanded to a Pashtun community in Nangarhar. History of conflict, and security issues meant low levels of trust within communities	*Acceptability:* While the intervention was acceptable in more progressive regions, in conservative areas it was problematic for men and women to sit together in community meetings to score health centres; the implementation team sought to circumvent by developing processes to feed women’s views into community meetings *Intention to adopt:* Not assessed (not individual-level intervention) *Coverage:* Not assessed (project remained on pilot scale until recently)
Context	Post-conflict environment, low levels of trust in public healthcare system. Balanced scorecard used at national level to monitor health services, therefore government officials were familiar with the use of scorecards in general	*Feasibility:* Initial substantial scepticism on the part of providers and local council members about the ability of the community to identify indicators and score performance, but their ability to do so was demonstrated over time
Scaling-up strategy	Vertical scaling up – influence MOPH to establish community scorecard as a national policy for community healthcare in Afghanistan	*Implementation cost*: Cost analysis not done, but recognised importance of highly skilled facilitators to effectiveness of intervention and need to train facilitators well *Sustainability:* Initial project documentation gave little role to Provincial Public Health Directors, but by end of Year 1 the project had learnt of their importance in coordination and was making efforts to work closely with them so as to facilitate institutionalisation

*FHS* Future Health Systems, *IR* implementation research, *MOPH* Ministry of Public Health, *NGO* non-governmental organisation

**Table 3:** Bangladesh: interventions, scale-up strategy and implementation research

Scaling-up dimensions informed by IR	Nature of intervention and scale-up strategy	Findings from IR
Implementing team	ICDDR,B in collaboration with a local for-profit mHealth company	
Research aim and design	Aim to assess acceptability of mHealth, the effectiveness of the intervention and user satisfaction Initially designed as quasi experimental study with intervention and control, employing surveys of informal healthcare providers (VDs), exit interviews, household survey and routine data; evolved into more observational design, given widespread adoption of mHealth schemes	
Attributes of the innovation or service	mHealth call centre to advise VDs and a self-diagnostic tool for use by villagers and VDs Aim to enhance the quality of care provided by untrained VDs	*Appropriateness:* Not assessed *Fidelity:* Not assessed
Attributes of the target community	Interventions focused in Chakaria, a remote rural area of Bangladesh hosting a health and demographic surveillance site, where the implementer ICDDR,B had long-standing relations	*Acceptability:* Seeking advice from formal doctors was perceived by the VDs to undermine their own capacity and threaten their reputations; clients preferred face-to-face consultations to telephone ones, and preferred to consult with known doctors; they had limited trust in services provided from a distance by unknown doctors *Intention to adopt:* A survey of the local population’s readiness to adopt mHealth [34] found that only 50% of the population who owned a mobile phone knew how to text, and only 5% used the internet; overall, there was a lack of trust in mHealth approaches *Coverage:* Not assessed
Context	Government policies very supportive of e-health; ICDDR,B had previously worked with informal healthcare providers in the area and had close relationships with them; rapid growth of e-health initiatives during the study period	*Feasibility:* Under the initial call centre model, a modest financial incentive had been set for the both the VDs and the call centre; given the low number of clients this proved too small to be meaningful to the call centre, leading to failure of the intervention
Scaling-up strategy	Spontaneous scaling up – multiple private sector actors would scale-up proven interventions themselves	*Implementation cost*: Not assessed *Sustainability:* Intervention as originally designed proved unsustainable given low demand, and lack of willingness of for-profit company to continue to provide services given low revenues from the scheme

*IR* implementation research, *VD* village doctor

**Table 4:** Uganda: interventions, scale-up strategy and implementation research

Scaling-up dimensions informed by IR	Nature of intervention and scale-up strategy	Findings from IR
Implementing team	Makerere University, in collaboration with local health system actors (health facility staff and district health management teams) and community actors, including local councils and savings groups	
Research aim and design	Aim to explore how mobilisation of community and other local stakeholders can enhance the delivery of quality maternal and neonatal health services in a sustainable fashion (feasibility and effectiveness) Designed as participatory action research, involving stakeholders in Susman’s action research cycle; draws on routine monitoring data (from health service, project documents), household and facility surveys, and qualitative methods, e.g. focus group discussions, in-depth interviews	
Attributes of the innovation or service	Multifaceted intervention to enhance maternal and neonatal health outcomes; included stimulating demand (through transport vouchers, radio sensitisation and CHWs) and improving quality of care (through training, non-financial recognition and supportive supervision) Aim: Enhance the use and quality of maternity and neonatal services at primary care facilities	*Appropriateness:* Not assessed *Fidelity:* Investments in training, supportive supervision and mentorship did not always lead to improved quality due to lack of drugs and supplies Quality of mentorship provided was quite mixed
Attributes of the target community	Implemented in three rural districts in Eastern Uganda: Pallisa, Kibuku and Kamuli; community members welcomed initiative but there were initial concerns about how sensitive local women, and particularly men, were to the need for antenatal care and attended delivery	*Acceptability:* Not assessed *Intention to adopt:* Stimulating demand for services was key to promoting adoption: active VHTs helped to spread understanding of the intervention among community members, as did strong political support from local politicians and community leaders *Coverage:* 48% of pregnant women in the intervention area were visited by a CHW at least once during the end line compared to 22% of pregnant women in the control; facility delivery increased from 63% to 73% in the intervention area while it remained almost stagnant in the control (64% vs. 63%)
Context	Previous FHS work in the same districts had introduced a successful maternal health voucher scheme but had struggled to financially sustain this; focal districts characterised by considerable poverty and relatively remote rural communities with weak infrastructure, there was concern about the sustainability of the intervention	*Feasibility:* Recognised (1) extensive supportive supervision to CHWs and district implementation committees were needed to implement intervention well; (2) use of existing structures, such as savings groups and CHWs, was found to be effective; (3) payment for vouchers via mobile money schemes was complicated given the multiple processes involved and therefore took significant investment of time and effort to set up; (4) cost of voucher scheme was prohibitive; (5) danger of theft of savings accounts in rural areas without banking
Scaling-up strategy	Diversification – sought to encourage and engage Ministry of Health and other actors at national and district level to add or continue to include effective elements of Makerere packages in their own interventions	*Implementation cost*: Cost analysis not done, but team devised lower cost implementation strategies, e.g. used “Super VHTs” (i.e. high performing VHTs to train other VHTs), also sought to support some community savings groups to train others *Sustainability:* The use of local stakeholders and structures was found to be critical for buy-in, ownership and continuity; local stakeholder commitment was central to sustaining project strategies such as performance review for better service delivery Continued stakeholder engagement was found to be key to dealing with day-to-day challenges of service delivery and improved relations among stakeholders, but also heightened the level of responsibility at individual and structural levels

*CHW* community health worker, FHS Future Health Systems, *IR* implementation research, *VHTs* Village Health Teams

### Attributes of the intervention

While all three of the country initiatives sought to improve the quality of health services available to the rural poor, the intervention strategies employed across the three settings were quite different. The community scorecard in Afghanistan was thought by the research team and local stakeholders to be an appropriate intervention given the high levels of existing mistrust within communities, and particularly between communities and authorities (including health workers). The community scorecard was perceived as a means to engage multiple stakeholders, including provincial and district directorates, non-governmental organisations (NGOs), healthcare providers, councils and communities. At the same time, it was intended to strengthen social accountability and audit of healthcare services, enhance community governance and engagement toward state building, and build trust in a post-conflict environment. Further, it built upon an established system of balanced scorecards that had been employed nationwide to track the performance of health facilities and hospitals. The intervention in Afghanistan took place over a shorter period than those in Bangladesh or Uganda, and there was relatively limited evolution of the intervention during the FHS project so the intervention, as originally conceived, closely matched the final form of the intervention implemented by FHS; however, the intervention evolved after FHS had formally closed out its work. Specifically, an NGO was asked to test the same intervention in a different ethnic community, and after the successful conclusion of this, in 2015, the government passed a national policy mandating the community scorecard as a key strategy for all NGOs delivering the Basic Package of Health Services, and by 2017 the intervention was being implemented in five different provinces.

In Bangladesh, the project team started with a more fluid formulation of the intervention noting that they sought to support informal healthcare providers (VDs) in the intervention area through the provision of technology resources to formally trained healthcare providers or of decision guides. Initially, the project team planned to adopt two interventions, one, known as the ‘Health Box’, was intended as a self-diagnosis and treatment guide for communities, which could also be used by VDs, and the other was the telephone link to a medical call centre run by a private for-profit partner. The Health Box technology was dropped during the first year as it was found to be designed to assist in diagnosis and treatment of specific diseases such as HIV/AIDS and was not easily adaptable for a wider array of conditions.

The Bangladesh project team focussed their efforts on technologies to support VDs, and the call centre was launched in mid-2011. The call centre services were provided for a fixed rate fee set by the private for-profit firm. Unfortunately, by the end of 2011 it was clear that this system was not working. The number of clients using the call centre was low due to low levels of awareness and trust, resulting in insufficient financial gain for the firm to continue its services. Hence, the private for-profit firm lost interest in running the call centre and became unresponsive. Further, communication between patients and the call centre doctors was difficult due to the different dialects spoken. Subsequent to the withdrawal of services by the for-profit firm, ICDDR,B decided to launch its own call centre, building on what it had learnt from the earlier collaboration. At the time of writing, the ICDDR,B call centre is still functional but has not significantly scaled up its operations.

The FHS Uganda team consciously adopted a flexible intervention design, initially tentatively identifying a package of related interventions to improve the use of maternal health services but committing to work with stakeholders during the early years of the project through a participatory action research approach in order to refine the intervention. While, throughout this process, the three main pillars of the intervention remained the same (promoting community demand for maternity services, supporting improved quality of care, and enhancing access to services through measures to address transport costs), the actual intervention details changed substantively through the process. In particular, the use of both transport and healthcare vouchers were dropped early in the process as being both financially unsustainable and too logistically complicated to implement. Instead, the team sought to work with local savings groups to help women save funds to cover the costs of their own maternity care, and also instituted a system of recognition awards as a low-cost way to motivate health workers. While the interventions as implemented by the FHS team have not been fully replicated elsewhere, the interventions have been sustained by local actors and aspects of them continue to influence broader government policy.

### Scale-up strategies

The three different country teams each adopted very different strategies to scaling up their interventions; however, none of the teams articulated clear scale-up plans in their initial proposals. The Afghanistan team recognised relatively early in the implementation process that it sought to stimulate a change in government policy towards the use of the scorecard, and this insight was important in terms of the implementing team established and in particular the strong links built by the research team with the Department of Community-based Health Care in the Ministry of Health. However, the Afghan Government was not initially convinced by the results of the community scorecard intervention, observing that the initiative had not included Pashtun communities (only ethnic Hazara, Uzbek and Tajik communities) and that before seeking to scale-up, it would be important to implement the community scorecards in contexts that included Pashtuns. It was only after this further piloting, which was implemented with another NGO in Nangarhar province after the formal end of the FHS project in Afghanistan demonstrating similar evidence of improvements in health facility services, that in September 2015, the Ministry of Public Health gave official support to the community scorecard strategy incorporating it as part of the new Community-based Health Strategy.

In Bangladesh, the FHS implementing team quickly recognised (by the end of 2011) that there was an explosion of mHealth technologies with many different organisations experimenting with different mHealth applications. The FHS team sought to better understand the array of different mHealth models being implemented, through conducting a scoping study [24]. Of the many mHealth applications found, only the FHS initiative and one other social enterprise were seeking to connect VDs with formal doctors. However, the team’s awareness that there were many other mHealth initiatives, and that most worked through the private sector, perhaps influenced the team to consider a more spontaneous scaling-up strategy that focused on identifying successful aspects of different models, and allowing the market to adopt such innovations.

While the FHS Uganda team did not articulate a clear scale-up strategy at the start of its work, the broad approach adopted did not hinge upon an organised replication of the scheme. Instead, the FHS Uganda team sought firstly to design an intervention that could be managed and sustained by local communities and stakeholders themselves, and secondly to build relationships with a variety of stakeholders, including the Ministry of Health, parliamentary representatives, NGOs and development partners, so as to encourage them to adopt and roll out successful elements of the FHS approach.

### Actors: attributes of the target community and implementers

All three research teams conducted a stakeholder analysis at the start of their work, although these were conducted with varying degrees of thoroughness. In Uganda, the stakeholder analysis suggested that the community was very supportive of the intervention, though concerned about feasibility considering local resource constraints [25]. The question of community acceptability was a greater concern in Afghanistan, where the team identified very strong support from the Ministry of Public Health from the outset, but low levels of trust, linked to the current and historical security situation, and particularly strong mistrust within communities towards public authorities. Further, the implementing team was initially concerned about the feasibility of ensuring representation in the community scorecard discussions of the full diversity of community members. However, the final round of focus group discussions conducted in the intervention communities found very high levels of participation and a sense that even the most marginalised community members had been involved in the process, ensuring gender equity and representation in the generation, scoring and management of performance indicators.

All three of the research teams identified a complex array of stakeholders that they needed to engage (Table 5) to support both the successful implementation of the intervention and subsequent scale-up. In practice, quite a lot of the research conducted, and especially ‘add on’ studies developed after the main protocols, focused heavily on understanding the attributes of stakeholders. For example, the Afghanistan study used focus group discussions to explore which community members were involved in the community scorecard processes, and how they perceived the process. In Bangladesh, research addressed how VDs perceived the call centre intervention [17], and later spread into studies of citizens’ use of mobile phones [19], with a view to understanding how the technology could best be used to promote health. In Uganda, the participatory nature of the research meant that the research team had very close engagement with all the stakeholder groups listed in Table 5. For example, throughout the implementation process, the team conducted regular review meetings with an array of local stakeholders involved in the project to review which parts of the intervention were working smoothly and which were facing obstacles. Ultimately, the team identified key findings for all the key stakeholder groups (village health teams, communities, savings groups, health workers, political leaders, health managers and district administrators).

**Table 5:** Relevant stakeholders identified across the three countries

	Afghanistan	Bangladesh	Uganda
Community level	Local community leaders and health councils – support for the scorecard programme	Village doctors – main target of intervention	Village health workers (village health teams) involved in implementing the intervention
	Local NGOs that helped to implement the project	Villagers, as users of the proposed mHealth technologies	Health facility staff, including officers in charge
			Savings groups, involved in helping households save for deliveries
District and provincial level	Provincial Public Health Directors important in terms of coordination and providing support to facilities		District local councils, district health officers and sub-county councils
National	Community-based Health Care Department at Ministry of Public Health, which became the champion for the community scorecards	Technical Advisory Group for ICT in health (formed by ICDDR,B during 2014)	Ministry of Health, especially the Reproductive Health Division and the Planning Department
Private sector actors		Private for-profit firm that initially provided the call centre service	
		Other projects and companies running mHealth interventions in the same regions	

### The context

Reviewing the project documentation across the three sites, there is surprisingly little explicit recognition or discussion of contextual factors that may affect scale-up. While teams invested significant effort in actor management, and nurtured relationships with local and national level actors whose support they perceived to be critical to the success and ultimately scale-up of the intervention, there is much less analysis of the context.

There were some partial exceptions to this. First, in Afghanistan, the unfolding security situation meant that the team provided regular assessments and reports on the security risks, but other dimensions of context were not fully studied. In Uganda, community engagement through the participatory action research identified factors important for continuity of interventions, including local political ownership of the intervention, building of local capacity and committed leadership from both the political and technical arms of the districts in which the intervention was implemented.

One explanation for the limited systematic analysis of context may be the fact that, in all three countries, the research teams were local and/or had long histories of engagement in the regions where the interventions were occurring. For example, Makerere’s work in the Eastern region of Uganda dates back to the beginning of FHS1 (around 2006), Johns Hopkins School of Public Health involvement in Afghanistan dates back to 2002, and ICDDR,B has been working in Chakaria promoting self-help for health since 1994 [26]. It is possible that, given these histories of extended engagement in the intervention sites, teams were already intimate with contextual factors and did not feel a need for further in-depth investigation of context.

### Implementation research design and findings

In both Afghanistan and Uganda, the planned research focused primarily on the questions of feasibility and effectiveness, namely the extent to which the intervention could be carried out in that particular setting and whether or not it was effective. The Bangladesh team sought to examine both effectiveness and acceptability of the intervention.

Many of the findings from the implementation research studies related to the intervention itself, and in particular its acceptability, appropriateness, feasibility and implementation costs. These findings bore significant implications for the design and implementation of the intervention. For example, in Afghanistan, the studies underlined the importance of investing sufficiently in skilled facilitators who could inform communities about entitlements and mitigate unrealistic expectations. In Uganda, the implementation and documentation process pointed to the need for supportive supervision for CHWs and district implementation teams. In Bangladesh, the whole intervention design was brought into question as it became apparent that the intervention, as initially conceived, struggled to achieve acceptability with community members, and did not offer sufficient financial incentives to either the village doctors, or the private for-profit call centre.

In addition to lessons about the design of the intervention, the research also yielded insights into how stakeholders perceived the intervention and how best to frame and implement the intervention to promote its acceptability and perceived appropriateness. For example, in Afghanistan, in conservative communities, it was important to figure out modalities that allowed women’s views to be fed into the community consultative processes, even though, in some settings, the women themselves were unable to participate alongside the men. Further, the research team realised that local stakeholders found the intervention more acceptable when framed as part of a national system of cascading scorecards, rather than as a separate and stand-alone intervention. While all three of the country teams pursued qualitative research, such as focus group discussions, which could help cast light on acceptability and appropriateness of the intervention, many important findings emerged spontaneously from the research team’s close and regular engagement with stakeholders on the ground.

Frequently, the original objectives of the implementation research undertaken by FHS appeared unnecessarily narrow. While research protocols focused on feasibility, acceptability and effectiveness, the close involvement of the research teams in these implementation processes also cast light on factors such as cost, fidelity, sustainability and the perceived appropriateness of the intervention. These insights offered important and actionable lessons for implementing teams.

## Discussion

### Contribution of implementation research to scaling up

While there are obvious connections between the conceptual frameworks on scaling up [8, 9] and frameworks on implementation research [15], to the best of our knowledge, this is the first paper that explicitly integrates these two types of framework.

Although the projects presented here are only now reaching completion, and it is therefore too early to fully assess their influence upon scale-up and sustainability, the research conducted offered substantial insights into the barriers and facilitators of scaling up, including the design of interventions, and relevant attributes of stakeholders. Frequently, these insights could be translated into concrete changes in the design or framing of an intervention that might make it more feasible or acceptable. The research process in all three cases involved substantial stakeholder engagement, which, while burdensome on the research team, was critical to both developing lessons and encouraging the adoption of these lessons.

In both Bangladesh and Uganda, the interventions were consciously framed in a relatively flexible way that facilitated adaptation to new learnings about stakeholders, or the intervention design. This flexibility was of critical importance, and all three FHS projects went through substantial adaptations to the initial design, including dropping some elements of the original package of interventions (Bangladesh and Uganda), adding new elements to the package of interventions (Uganda), reframing or re-packaging the intervention (Afghanistan), changes in implementation partners (Bangladesh), and extending the time frame and the geographical spread of the intervention (Afghanistan). Other authors have pointed out that scaling up interventions or innovations involves intervening in complex systems [5, 27], such that one should expect constant adaptation, dynamic interactions and complex social processes. Certainly, the three case studies reflect this complexity, and the implementation research conducted offered a means to embrace the complexity and simultaneously navigate a way through it. It is also important to note the potential tension between adaption and fidelity. While fidelity of an intervention to its original design is often thought to be key to the success of scale-up, this needs to be balanced against adaptations that help ensure the appropriateness and acceptability of the intervention. Particularly in the early stages of intervention design and scale-up, adaptation will likely be more important than fidelity.

### Characteristics of effective implementation research for scaling up

There were considerable differences in the implementation research designs adopted across the different country settings. While all three country research teams sought to assess effectiveness, they had differing foci in terms of other implementation questions (feasibility, acceptability, etc.). Further, there were differing degrees of flexibility in the research design, with Uganda perhaps being the most flexible and adopting a participatory action research approach, and Afghanistan, for primarily logistical reasons (namely the shorter period of study), being the least flexible. However, in addition to the formal protocols, all three teams were required by the FHS research consortium and the funders to also develop theories of change [28] and conduct stakeholder analyses early in the research process. The stakeholder analyses proved critical in identifying key stakeholders, and guiding teams’ thinking about how best to engage those stakeholders. Further, the theories of change necessitated an explicit articulation of how the proposed interventions would help achieve the desired objectives, and therefore perhaps facilitated later decisions about which components of an intervention could be dropped or replaced. Overall, these pragmatic project management tools combined effectively with the implementation research to enable teams to adapt implementation strategies to reflect learnings from their research, while maintaining an overall sense of purpose and strategy.

In Uganda, the Makerere research team also engaged in regular review and reflection meetings. To give one example, during the first year, participatory self-assessments were conducted where both the research team and the district health management teams involved in the intervention reflected on what they had learnt, the nature of their relationships, where there were gaps and how these gaps could be addressed. This process helped to identify where collaboration between the research team and the district health management teams could be improved, and where responsibilities needed to be handed over from the research team to district health management teams to strengthen effectiveness and sustainability. Such formal processes for reflection likely helped to reap the full benefits of implementation research.

The three cases reviewed here had varied approaches to scaling up, including a more vertically oriented approach (Afghanistan), a more horizontal approach (Uganda) and a replication approach (Bangladesh). In Afghanistan, the research focussed primarily on the feasibility, acceptability and appropriateness of the intervention. Presumably in this context, where government already used balanced scorecards at higher levels in the system, determination of feasibility and desirability may be sufficient to support scale-up. In contrast, the research in Bangladesh, where it was anticipated that private market-based actors may be key in scaling-up the intervention, focussed more on acceptability and adoption, i.e. attributes of the target community. In a sense, the Bangladesh study provided ‘market research’ for private sector entities interested in entering the market. However, these conclusions are tentative, and much more needs to be done to understand how best to tailor implementation research to different types of scale-up strategy.

### Study limitations

As previously noted, this paper is written at the time of the close out of the FHS projects, and thus it is too early to fully appreciate lessons concerning scale-up and sustainability. In addition, all members of the writing team are internal to the FHS project, and indeed the lead author is also the project leader of FHS. This has certain advantages in that, as participants in the processes described here, the authors brought extensive knowledge of the contexts, interventions and implementation research. However, it may also lead to certain biases, such as an inclination to make the interventions supported appear successful. We sought to guard against this bias as far as possible by triangulating between different data sources, including the researchers' own recollections, but also historical project documentation to describe the initial ambitions of the projects and how they changed over time.

### Implications for future research and practice

Many implications emerge from this paper regarding how best to design and undertake implementation research that is designed to support scale-up.

With regard to implementation research, a number of apparent good practices have emerged, many of which reinforce what has already been described in the published literature. For example, the need for engaging stakeholders from the start of the process and providing ongoing nurturing of these relationships is highlighted across multiple implementation research texts [29, 30], and was again found to be important here. Further, this paper also supports the need for flexibility in both the intervention being implemented (to respond to lessons from research findings) and in the research design (to respond to emerging new problems) [31].

While it is important that teams involved in implementation research are not overly ambitious in their objectives, teams should think more broadly about the kind of questions that their research can address, as well as being receptive to unexpected learnings.

Finally, while scaling up frameworks typically consider the characteristics of the target community and those of the implementing team separately, FHS research suggests that this is an oversimplification. There were multiple stakeholders involved in each of the country cases, who had differing degrees of responsibility for and engagement with the intervention. In no case was the community a passive recipient of the intervention. We also found substantial blurring between the research team and the ‘implementing team’. In all three of the cases considered here, the research team worked closely with stakeholders responsible for implementation, and had a direct implementation role themselves. In Uganda, where participatory action research was employed, community members were in effect part of the implementing team.

There is a growing interest in the notion of ‘embedded research’ [32, 33], whereby researchers are embedded within the organisations that they are examining. While only one of the three studies described here formally espoused a participatory action research approach, the close relationships, and indeed blurring of boundaries, across different types of stakeholder roles was of critical importance to the success of scaling up strategies. However, this relational complexity and the overlapping roles across research team, implementation team, and target community raise many questions around the usual research paradigm that emphasises the importance of research neutrality, and sees an intervention as largely separate from the community and context in which it is implemented. If the application of implementation research to support scale-up of interventions depends critically on intense stakeholder engagement, then more thought is needed about how best to manage these relationships through the scale-up process.

## Conclusions

Implementation research can provide significant insights into how best to scale-up a particular intervention, including offering learnings about the design of the intervention, the characteristics of stakeholders engaged in the intervention and the scaling up strategy. To take advantage of the promise of implementation research, scale-up strategies require flexibility, as does the design of implementation research intended to examine it. Project management tools, such as theories of change, and stakeholder analyses can be critical adjuncts to the research, ensuring that adaptations made to initial plans continue to serve overarching project objectives and take account of complex stakeholder landscapes.

## Open peer review

Peer review reports for this article are available in Additional file 2.

## Additional files

Additional file 1: Rich description of cases. (DOCX 155 kb)

Additional file 2: Open peer review reports. (DOCX 38 kb)

## Abbreviations

FHS: Future Health Systems
NGO: non-governmental organisation
VD: village doctor
