# Supplementary material for: Strengthening scaling up through learning from implementation: comparing experiences from Afghanistan, Bangladesh and Uganda
Source: Health Res Policy Syst. 2017 Dec 28;15(Suppl 2):108. doi: 10.1186/s12961-017-0270-0 (PMC5751808; doi:10.1186/s12961-017-0270-0)
Supplement: Supplementary file 1 — Rich description of cases. (DOCX 155 kb) [file 12961_2017_270_MOESM1_ESM.docx]

**Online Annex A – Rich Descriptions of Cases**

**FHS Afghanistan - Role of implementation research in scaling up the Community Scorecard**

***Attributes of the Innovation***

Original conception of the intervention was a Community Scorecard. Articulated outputs as being the identification of viable community communication and capacity building mechanisms. CSC involved repeated facilitation of community groups and interaction meetings with providers. Development of community level action plans, and repeated assessment of progress on CSC on a quarterly basis.

Original conception of intervention and final form of intervention did not seem to vary much.

***Attributes of the Adopting community***

- low levels of trust, linked to existing and historical security situation. Especially strong mistrust within communities towards public authorities. Team aware of this from start ie from protocol phase.
- In the inception phase identified one of risks being lack of representation of full diversity of community ie. exclusion of certain groups - however final FGDs found very high level of participation and sense that all different groups within communities, including most marginalized, had been able to contribute.
- Sought to engage local leaders in the CSC and secure their support.
- Initial selection of the communities in which the intervention would be implemented focused on the feasibility (ie. interest in/likely uptake of intervention as well as security related issues) and secondly linkages to pre-existing projects that were also experimenting with other types of community health service quality-related interventions. However in discussions with the MOPH during Year 2 of the project, the MOPH questioned the transferability of the project to other communities from other ethnic backgrounds (namely Pashtun and Tajik). The project therefore development plans to implement the intervention in another area Nangrahar working with a Dutch NGO called HealthNet, this Ngo was working with Pashtun and Tajik communities. This phase of work with a further NGO was later (Year 3) referred to as part of the “scaling up” phase – although initially there had not been a clear plan to roll out across other NGOs.

***Attributes of the Implementers***

Implementing team involved (i) the JHU team based in both Baltimore and Kabul as well as IDS team (ii) two local NGOs working in the two intervention provinces and (iii) the Community Based Health care department of the MOPH. (could be argued that this is a credible team involving international expertise – JHU, local expertise – Local NGOs and local recognized authority, and authority with responsibility for scale up ie. MOPH). Project maintained this close collaboration with the CBHC throughout, and recognized early on that sustained engagement with the MOPH was critical. FHS team members also provided TA to the CBHC thus further cementing this relationship.

The team invested quite heavily in capacity development for the facilitators responsible for facilitating the community meetings and at the same time took the opportunity to train other interested partners. For example, during Year 2 they provided training in CSC for 20 NGO managers, donors and MOPH officials. This included many of the individuals who had been interviewed as part of the original stakeholder analysis. Several of these individuals also attended the initial awareness-raising workshop where the concept of CSC was introduced. All 20 of these individuals were part of the Community Based Health Care (CBHC) network who therefore have major roles in technical advisory groups and working groups for CBHC

Year 1 report also talks about and discusses the need for close coordination with the Provincial Public Health Directors (PPHD). This was not observed in the initial documentation. But project started to make efforts to work closely with them.

***Attributes of the environment/context***

- Initially noted that there was already a balanced scorecard that operated at the national level, but this did not engage with community level services.
- Recognized security risks in environment and perceived this to be a major threat to the possibility of sustaining and scaling up the intervention. End Year 1 did have to drop one of the original 4 communities due to insecurity in that region.
- When PIRU officer visited, early Year 1 (November 2011) took opportunity to engage other bilateral actors eg. DFID, USAID & Aga Khan foundation around this agenda. Note also that became aware of SCF experimentation with Partnership Defined Quality Strategy – that is quite similar and the need to coordinate with this group.
- In December 2011 met with a JHPIEGO affiliate implementing the Health Sector Support Program to discuss their experience with the Partnership Defined Quality Strategy.
- Dec 2011 met with the QA team of the MOPH – rationale was that they did not want opposition from this group to MOPH support to scale up. *(note gradual awareness of how fill this policy environment is with related actors conducting similar work).*
- Jan 2012 – FHS team connected with the MOPH team with responsibility for harmonizing and aligning efforts to engage with communities – got a “seat at the table”

***Scaling up Strategy***

Always desired to institutionalize the scorecard through integrating into MOPH policies. Indeed goal identified early on as “change in government policy towards use of the scorecard”.

Wanted to pilot test in two different NGO sites in two different provinces (Bamyan and Takhar), and then convince national government of benefits leading to a vertical scaling up.

Govt not initially convinced, as noted above first asked about transferability of intervention to other communities of different ethnicities. Hence FHS team in year 3 began to work with other NGOs (notably HealthNet) in different regions with different ethnicities. At this point began to talk about voluntary uptake of CHC strategy as an alternative strategy for scaling up. However continued to work closely with CBHC unit within the MOPH. In 2015 (year 4)- after FHS afghanistan project was officially over, Dr Arwal in CBHC unit started advocacy for inclusion of CHC in national strategic plan. This finally came to fruition in Sept 2015 when Minister of Public Health gave official support to CSC as part of the new 2015-2020 Community based Health Strategy.

***Nature of the implementation research conducted***

- Initial stakeholder analysis to ascertain appropriate strategies for community engagement (KIIs)
- Originally planned to use existing data from NHSPA and RBF to supplement local data on service delivery. While the team sought to conduct this analysis, it was not as useful as anticipated because not all facilities were included in the survey due to the use of a stratified random sampling approach to selecting facilities.
- Wanted to use FGDs to explore issues such as trust in public institutions, perceptions of service access and utilization and how these changed over time. This was done.

***Lessons learned from the IR***

- One challenge identified early on (before implementation of the pilot began) was how to get around the issue of men and women not usually sitting together in public in this very conservative Islamic country. In the CSC process community members, health providers and other stakeholders, of all sexes, meet in the interphase meeting to discuss scores and fashion out an action plan together. It was thought that this might not be feasible in some communities and the team sought to explore ways of ensuring that the voices and input of women get carried through into action plans. Specifically, facilitators were trained to ensure that the viewpoints of the women were fed into and informed the final action plans. However, in Bamyan, an interesting phenomenon occurred, women demanded to be included in the male focus group discussion and ranking meetings, as they felt their perspectives and priorities may not be considered or valued otherwise. Thus in some facilities mixed gender discussion groups occurred.
- Recognized importance of couching the intervention in language that was familiar to stakeholders. For example, it helped to build upon the existing balanced scorecard that stakeholders within the MOPH were familiar with. The likely importance of this was recognized early in the project, but the experience throughout the project confirmed the point. Accordingly, during project presentations, the community scorecard was articulated as part of a set of “cascading scorecards” starting at the national level all the way down to the community – thus effectively integrating the CSC into the pre-existing BSC strategy.
- Recognized the importance of skilled facilitators for joint action planning and resource generation. Also recognized that these facilitators would not just naturally emerged, needed to be trained. Facilitators ability to get buy in to the intervention from all sorts of different groups within society was particularly key. Also facilitators need to be able to bring into balance community demands and expectations (ie. don’t have unrealistic expectations). Wonder whether facility councils might play this role in the future, in a scale up strategy.
- Perceived challenge to scaling up the CHC was the dynamic health system – with constant change eg. In CHW strategies, this made it harder for the CHC to fit in.
- “At the onset, supervisors, providers, council members, and other leaders expressed considerable skepticism about the ability of community members, a majority of who were illiterate to identify indicators and score performance. Evidence of results in less than three months (i.e. following Round 1) convinced the majority of these ‘skeptics’ of the potential of the CSC process to address critical performance issues and enhance community ownership and responsibility for the facility, including the delivery of care.” (Edward et al 2015).

**FHS Bangladesh – Role of implementation research in scaling up m-health initiatives in Bangladesh**

**Attributes of the innovation**

The Bangladesh team proposed a complex intervention/innovation that has evolved over time. The intervention is primarily concerned with linking formal and informal health care providers using novel forms of IT/m-health/e-health so as to address concerns that were previously identified (under FHS1) about the quality of care offered by informal health care providers known as Village Doctors (VDs).

Initially the research team started out by focusing on two technologies the Health Box (interactive software actually designed for community use) that allows self-diagnosis and treatment, but in the case of the FHS project was going to be used by VDs, and secondly a call center staffed by qualified doctors who were to provide diagnosis and treatment back up to VDs.

The Healthbox technology was dropped in Year 1 of the project, due to lack of support/breakdown in negotiations with inventors of the HealthBox, as well as recognition of the fact that it was designed to assist in diagnosis and treatment of specific diseases such as HIV/AIDS and was not easily adaptable for a wider array of conditions. Further at this time negotiations with TRCL the provider of the call in center were going well.

The TRCL call center was launched in mid-2011 (year 1 of the project) however by December 2011 the call center had become unresponsive, and the quality of support from the call center was undermining the implementation of the intervention. The intervention persisted for some time, but was eventually discontinued. The primary reasons (and learnings) were that (i) the financial incentives for supporting the call center were insufficient for the private for-profit firm providing the support and (ii) TRCL struggled to find doctors with appropriate language skills.

Subsequent to this, ICDDRB decided to launch its own call center, based on what it had learned from observing TRCL operations. As a non-profit entity ICDDBR was not seeking to make a profit, and it had experience working in the relevant areas and doctors with relevant languages. The ICDDRB call center was launched in 2014, and in June 2014 video conferencing via skype was added.

**Attributes of adopting community**

FHS implemented a number of qualitative and quantitative studies that focused on the attributes of the adopting community in terms both of the Village Doctors (this was largely qualitative) and the community members (both qual and quant studies.)

These studies revealed the following:-

- From the VD perspective, the link with formal health care providers discouraged prescription of too many drugs (which was a good thing)
- Seeking advice from formal doctors was perceived by the VDs to undermine their own capacity and threaten their reputation.
- Under the initial model run by TRCL VDs were concerned that the financial incentive that remained with them when they consulted the call center was too small to be meaningful.
- Clients stated that they preferred face-to-face consulations over tele-consultations.
- Clients prefer to consult with known doctors, and they had a limited degree of trust in services provided from a distance.

A paper by Khatun et al (published in 2015) provided more quantitative findings. For example only 50% of the population who owned a mobile phone knew how to text. (and this was also inhibited by low community literacy). And only 5% of the those who owned a phone used the internet on it (despite widespread availability of cheap smart phones). Most important a majority of people expressed concerns about trusting m-health and the quality of care provided through m-health initiatives. Further ownership and access to m-phone technology was inequitable and significiantly lower among women, the poor and older adults.

**Attributes of the Implementers.**

ICDDRB was the primary research group, but was also involved in implementation. Its partner in implementation was TRCL. This was a Bangladesh based m-health provider with call centers in Bangladesh and throughout the middle-east. TRCL was a private for-profit provider, and the FHS team recognized the potential conflict that this for-profit/non-profit collaboration could bring from the beginning. While TRCL was initially prepared to forgo profits on this venture as a kind of CSR initiative, this position did not last long. It is not clear how TRCL’s expectations differed from what actually unfolded…perhaps other priorities arose, perhaps they had expected a more dramatic take off…the reason for the company’s declining interest in the project is not clear.

ICDDRB is obviously a well known and well respected research group and service provider in Bangladesh. It also has close ties to policy makers. Further, it had worked closely with the VDs in the intervention area, during the former round of FHS1.

ICDDRB became aware of the many (often competing) mhealth initiatives in the country and within the institute and decided to form a TAG for ICT in health (during 2014 – Year 3) of the project. This TAG brought in outside advisors (both academic and policy makers including from the Ministry) and sought to align different mhealth research work, as well as develop a stronger focus on equity and accountability.

**Context**

FHS/ICDDRB had experience from FHS1 that identified harmful practices of village doctors and tried to intervene through providing training, but this was unsuccessful, especially as loss of income was a barrier.

While FHS was working on its intervention there was an explosion of e-health and m-health initiatives in Bangladesh (year 1) - FHS had sufficient flexibility to switch track somewhat and conduct a scoping study of these initiatives.

E-health policies in Bangladesh are very supportive of m-health (need to check to see if FHS played any role in influencing this).

**Scaling-up strategy**

FHS strategy was to find a successful model and then publicise this and leave it to the market to be replicated. This was part of the appeal of working with TRCL.

In practice we found rapid prototyping and rolling out of different m-health interventions in a fairly haphazard way …so FHS research perhaps had a role in shaping a scale-up that was already going on.

**Nature of research**

- Wanted to assess acceptability of telemedicine and health box to VDs and community, and also assess effectiveness.
- Originally planned (i) analysis of MIS data generated through TRCL system, (ii) household surveys as part of regular and HDSS surveillance (eg. Covering illness, and source of care as well as somequestions about telephone use) and (iii) qualitative data from FDGs and IDIs with VDs and community members.
- Ended up adding on a scoping study of telemedicine as they sought to understand the breadth of what was going on.

**FHS Uganda – MANIFEST**

**Attributes of the Innovation**

The intervention in Uganda was designed to reduce maternal and newborn mortality and morbidity through improved quality of care and increased utilization of maternal health care services.

The intervention design grew out of previous work conducted by the Makerere University team in the same districts, which had piloted the use of vouchers for local transport providers to help get women to maternity care services. This previous voucher scheme had proved very successful, to the point where it became challenging to figure out how to financially sustain the scheme. Accordingly, the new intervention and study pursued during the period 2011-2016 focussed on a broader package of interventions that used community resources to support the transport vouchers. Specifically the initially envisaged components of the intervention included:-

- A community awareness campaign – through radio, workshops and publications designed to educate people about the importance of maternity care, and enhance their awareness of transport vouchers
- Ongoing provision of transport vouchers – though perhaps for a more limited group of clients so as to increase affordability, and with the community picking up the responsibility for financing the vouchers
- Provision of health care vouchers
- Supportive supervision and training for health workers to enhance their skills
- Improved supply of drugs and equipment

This intervention was to be implemented in two districts in Eastern Uganda, and to be elaborated and explored using a participatory action research approach. A third district was later added to the intervention area.

The team were concerned from the start about sustainability and explicitly wanted to design a voucher scheme that was simple enough for local level community actors to manage and to pay for. Accordingly, they planned to investigate alternative approaches for managing payment of vouchers, including mobile money schemes, and involving stakeholders such as health unit management committees and CHWs in managing funds, as well as building on local existing financial social networks such as burial groups and village savings and loan associations.

By June 2012 there was greater emphasis on motivating health workers eg. through giving them awards and designating centers of excellence. But while the protocols and plans, & IRB clearances for MANIFEST had been done, data collection had not yet started and did not start until the end of 2013. This slow start up presumably reflects time devoted to sensitizing stakeholders on the ground, developing materials (eg. for training, radio slots) and baseline data collection. Ultimately, the interventions included in the package comprised:-

- mentoring and supportive supervision for health workers and facility managers,
- a health worker recognition scheme to reward good performance
- community sensitization (home visits by CHWs, community dialogue meetings and radio sensization programs)
- promotion of saving through saving groups
- provision of transport using local transport providers.

Early findings (end 2014) focussed mainly on the importance of strengthening management skills. Training encompassed aspects such as training district health teams in skills such as conflict resolution, stakeholder engagement and team work. The transport vouchers were completely dropped from the final design of the MANIFEST project, as they were felt to be unaffordable. Instead the team focussed on promotion of savings through savings groups. Further while initially consideration was given to vouchers for health care, this was also dropped, and instead the team focussed on non-financial incentives for health care providers. While the savings groups were perceived to hold promise in terms of promoting sustainability, they were also found to be small and poorly organized. Thus the implementers worked with community development officers, a cadre of government official, to help strengthen the savings group and train the leaders of such groups.

The whole project adopted a participatory action research approach, drawing a lot on community insights to identify problems and help frame interventions.

**Attributes of the Adopting community**

Local communities were supportive of the intervention from the start – mothers appreciated the opportunity to deliver in health care facilities, with skilled birth attendants, and men were also found to be supportive, largely because the program appeared to help them with household financial management (Namazzi et al stakeholder analysis). Limited resources, especially financial resources, at the local level were felt to be the largest barrier to pursuing the intervention – especially as the implementing team were clear that the intervention had to be implemented in a financially sustainable fashion.

Savings groups are existing structures within the community, but the research team found that they were frequently small, poorly managed and tended not to promote savings specifically for health although they could help occassionally if there was an emergency. Part of the intervention (see above) involved providing community development officers to strengthen these savings groups. By the end of the project there were 1006 such savings groups in the three districts where the project was working.

**Attributes of the implementers**

There were extensive efforts to engage and involve multiple actors. Given the participatory action research approach used by the project it is frequently difficult to distinguish ”community” from “implementers”.

The team recognized the need to involve implementers from the start. In addition to the wide array of local stakeholders from community, facility and district level (described below) they also sought to get support at national level by involving two senior officers from the reproductive health division and Planning department of the Ministry of Health, as well as working closely with District Health Officers and District local councils. The stakeholder analysis conducted at the beginning of the project found that MOH staff were skeptical about the program that Makerere had been supporting, largely because they did not view financial vouchers for transport to be sustainable^[[1]](#footnote-1)^.

Year 1 (2011) annual report states that: FHS Uganda had met with the Speaker of the House during debates on funding for the Department of Health, has introduced the project to President Museveni, and has engaged members of the Ministry of Health on ways to scale up a voucher scheme to improve utilisation of maternal health services. The project also leveraged contacts with the MP of one of the districts where they were working to secure high level access to other policy makers such as parliamentary committees.

In 2013 a presentation about all FHS studies was made to the Uganda Maternal and Child cluster meeting at the Ministry of Health Headquarters. In addition the Uganda team convened a one day symposium with the Ministry of Public Health surrounding Safe Motherhood Week.

By the end of 2014 - the FHS Uganda team had had multiple meetings with stakeholders at district and sub district levels to publicise the importance of addressing issues related to maternal health through a multi-sectoral approach. The team noted that this had raised the profile of maternal health and emphasised the importance of involving a diversity of stakeholders in the fight to save mothers and newborns. It had also contributed to increased intersectoral collaboration, for example the sub-county leadership is contributing to solving problems such as lack of placenta pits and lighting at the health facilities.

A very broad set of local stakeholders were involved in the work, for example, a 2013-14 monthly monitoring report notes: we plan to sensitize local council leaders (LC 1) at the community level so that they support the VHTs during mobilization of the communities. We plan to involve the district development officers, the sub county committees, local council leaders more in monitoring MANIFEST project work because they interact closely with the people we are trying to reach and so could help to ensure project objectives are implemented. The project sought to hold quarterly meetings across all districts to provide support to local implementers, and on top of this also provided a substantial number of trainings.

The implementation team were highly reflective about how they approached implementation. This appears to have been strengthened by outside support from JHU. For example during year 1 the team completed participatory self-assessments where both the research team and the DHMTs reflected on (i) what they had learnt (ii) the nature of their relationships and where there were gaps and how they could be improved. This process helped to identify where collaboration between the research team and the DHMTs could be improved, and where responsibilities needed to be handed over from the research team to DHMTs.

**Attributes of the environment/context**

There is surprisingly little in the reports and documentation about the broader context and how it changes.

A 2013 annual meeting report comments on the change over in staff at two of the districts. It was noted that even if new staff coming in were good, it still takes a long while to bring them on board and orient them. By the end of 2013 there had also been political changes with some MPs leaving their posts and being replaced by others in elections. The research team noted that this meant they had to re-sensitize these local politicians to get them on board.

**Scaling up strategy**

There does not appear to be any intentional, planned scaling up strategy. Rather the implementation team were keen to learn relevant lessons that could be adopted by a multiplicity of other actors. They also sought to implement the intervention in a way that local communities could sustain it.

2013 FHS annual meeting – one of the lessons learnt – sought to put communities and district leadership “in charge” of the interventions – for example getting them to explain to other stakeholders what the intervention is about, has helped ensure a positive reception for the intervention.

By December 2013 it was noted that other development partners in Uganda have become interested in the idea of transport vouchers, specifically the Baylor College of Medicine Children's Foundation, Uganda, is implementing a transport voucher project in the districts of Kabarole, Kamwenge, Kibaale and Kyenjojo to transport pregnant women to the health centres. CUAMM Uganda is designing a transport voucher scheme for MCH to serve 7 districts in Karamoja region. Learning from the FHS team have informed the designs used for the voucher projects for both of the above programs. Meetings were held with the program design teams for the new projects and FHS shared its experiences with them. The Ministry of health is also scaling up a voucher program in Eastern Uganda and lessons from the FHS voucher project and other voucher projects in the country have informed the current design of the voucher program.

June 2014 annual meeting – added new research question: What are the potential pathways for scaling-up and sustaining the innovations? Though it does not appear that this question was ever answered very directly. Also, at this time, challenges noted related to finding funding to scale up the intervention.

By end of 2015 the research team had observed that local actors were beginning to budget for some of the practices that the research team had been supporting, so as to enhance prospects for sustainability. For example sub counties were beginning to budget for some facilitation for the VHTs and CDOs so that they continue with the good work after Manifest has phased out. The sub counties in Pallisa and Kibuku also made arrangements to purchase motorcycle ambulances from their local budgets because they appreciated the importance of having access to transport for referral. This is as a result of various meetings with the sub county implementation committees. Further two districts, Kibuku district and kamuli district, had started having recognition meetings to recognize best performing health workers.

**Nature of the Implementation research conducted**

Initially the team committed to conducting baseline and endline household surveys and then complementing this with qualitative work during the whole process including Focus group discussions, in-depth interviews, facility surveys, participant observation etc. In fact it seems that a lot of the actual data collection focused on documentation during the process (eg. minutes from meetings), as well as observations.

As noted this was defined as participatory action research, following Suzman’s cycle of diagnosing the problem, planning action, taking action, evaluating and learning. The team planned to start with a very wide array of stakeholder meetings in the two districts at community, district and county level.

**Lessons learned from the IR**

The stakeholder analysis conducted at the beginning of the study identified a number of challenges that stakeholders anticipated to be associated with the proposed intervention. This included factors such as inadequate sensitivity on the part of local women and especially men, to the need for ANC and attended delivery, poor quality of health care, challenges with transport for referral services, poor physical infrastructure (roads, lighting) that affected ability to access services and the big question of sustainability. However the research that ensured gave much greater specificity to these challenges, and also identified a series of new lessons including:-

- Need for extensive supportive supervision to CHWs and district implementation committees so that they fully understand the intervention, and implement it well (2013).
- Active participation of the community through the Village Health teams so as to ensure better understanding of the intervention among community membrs (2014)
- Super VHTs can be very helpful in providing support to other VHTs. Also interested in other notions of peer support groups eg. between Savings groups and VHTs (presumably an economical way to build skills and scale up)
- The team frequently used a tiered approach to scaling up within the intervention districts eg. for the savings groups the team decided that they would work with a small number so that they became model savings groups and could then provide advice to other nearby savings groups.
- Encountered difficulties to resolve health systems constraints (eg. lack of drugs, staff shortages) in a project of this nature.
- Participatory approaches can help train and empower local implementers eg. health facility managers and DHMTs
- Potential for theft of savings accounts set up in rural areas increased since the saving groups saved more money than they did before and they were not able to lend out all of it or to invest all of it. The team needed to encourage the use of banks and investment so as to increase earnings from the money saved.
- The mentorship scheme appeared effective, but there were specific challenges eg. the quality of mentorship varied significantly across mentors, many health workers saw the mentorship scheme as an added burden rather than a help (but likely because of significant documentation involved), the team also concluded that they needed to use more local mentors. The mentorship team comprised of mentors from the district and the Association of Obstetrics and Gynaecology. Further, the team had hoped to provide mentorship to health staff in all facilities but had to scale this back to just 4 facilities per district given the intensive resource demands that good mentorship implied, the lack of suitable local mentors and the desire to at least have an impact in some facilities.
- The involvement of stakeholders such as politicians and other administrative and technical staff was useful in harnessing and mobilizing more resources. Eg. politicans were influential in mobilizing communities to undertake particular health interventions and influencing positive behavioural changes…. before the project engaged politicians it had been difficult to get community members to participate in community meetings.
- There were important and positive synergies between the three main components of the intervention – but the team also noted that they were not always able to bring about the improvements in quality of care that they were looking for due to factors outside of their control.
- Positive spillover effects from the savings groups – they help with savings not only for maternity care, but also for other unpredictable and costly events. They are now also being seen as a possible springboard for health insurance, since they can enable households to save money that can be used to pay health insurance premiums. Uganda is in the process of introducing a national health insurance scheme.

1. Although it should be noted that more recently, since the end of the FHS MANIFEST project, the Ministry of Health has been working with the World Bank to scale up vouchers for delivery services, that are targeted towards the poor. [↑](#footnote-ref-1)
