# Supplementary material for: Strengthening scaling up through learning from implementation: comparing experiences from Afghanistan, Bangladesh and Uganda
Source: Health Res Policy Syst. 2017 Dec 28;15(Suppl 2):108. doi: 10.1186/s12961-017-0270-0 (PMC5751808; doi:10.1186/s12961-017-0270-0)
Supplement: Supplementary file 2 — Open peer review reports. (DOCX 38 kb) [file 12961_2017_270_MOESM2_ESM.docx]

**Title:** Strengthening scaling up through learning from implementation: comparing experience from Afghanistan, Bangladesh and Uganda

**Reviewer 1:** Kara Hanson

**Reviewer’s report**

**General Comments**

This is a nicely written article which sets out to integrate frameworks for studying the scale-up of interventions and implementation research in order to explore how implementation research can inform scale-up. It applies these frameworks to three case studies of implementation of health system innovations in Afghanistan, Uganda and Bangladesh, drawn from the Future Health Systems RPC. The case studies draw on project records to develop thick descriptions of the interventions, the scaling up strategies and how implementation research informed scale-up.

The stated objectives of the paper are to:

(i) analyse how implementation research contributed to understanding of the attributes of the service or innovation being scaled up, the scaling up strategy, the actors involved and the context, and thus informed the overall process of scaling-up;
(ii) reflect on the characteristics of implementation research strategies that make them best suited to support scale up and sustainability, and how this may vary for different types of scaling up strategies.

In general the paper is well argued and well evidenced. My main comments are:

(1) I was not convinced that these two different conceptual frameworks (attributes of / factors influencing scale-up and IR questions) are really “integrated” as claimed in the background section of the abstract, or whether they are really two different frameworks, applied separately to “work through” each of the three interventions looking to identify similarities and differences. (indeed, the tables presenting the results on scale up and IR are kept separate). It would be good to have further clarification on how this analysis really integrates these frameworks – and have this a more prominent part of the discussion section, so that the original contribution of the paper is more clear
(2) I did not feel that the analysis addressed how variations in scale-up attributes had informed/influenced different IR strategies, and I would like to see a bit more analysis of this, so as to come to a clearer understanding of the second point in objective (ii) – ie. how scale-up strategies could/should/would influence IR strategies.

**Major Compulsory revisions**

Address points 1 and 2 above.

**Minor essential revisions**

Note that reference 9 is incomplete.

**Declaration of competing interests**

I declare that I have no competing interests.

**Reviewer 2:** Peter Fajans

**Reviewer’s report**

**General comments**

The manuscript addresses an extremely timely and important issues in global health today- that of how to best to strengthen the planning for and the process of scale up of health interventions/innovations in the context of weak health systems- and how to ensure that the gains achieved are sustained. There has been an emerging literature on this topic over the last decade and the recent attention to implementation research provides an opportunity to strengthen the design and implementation of pilot/demonstration and other programmatic research to test health interventions on a limited scale, but at the same time to provide the necessary learning to support future successful scale up and long term sustainability. There is also a need for more comparative country case studies of this process.

The manuscript is extremely well written and easy to read- a pleasure, which in some places tends to hide some significant shortcomings or areas of need in strengthening in the paper. These are discussed in detail in the attached review comments.

**Major compulsory revisions**

The primary compulsory revision is that there is a need for greater clarity in what the paper is actually discussing. The title and particularly the text in the first half of the manuscript seem to suggest that the paper is comparing the process of scaling up and sustainability of the interventions. Only when the reader reaches the discussion section does it seem to become clear that in fact the three projects appear to have ended prior to any systematic scaling up of the interventions, much less assessment of the actual sustainability of the interventions- whether on a initial small scale or following scaling up. Without this clarification the reader interested in scaling finds the lack of information about the development of the scaling up strategy or in depth discussion of factors related to sustainability to be problematic.

The authors should more directly (but briefly) discuss the potential bias resulting from the lead author and the key country researchers involved in the projects reviewing the project reports, synthesizing rich descriptions which then serve as data for the comparative analysis. I think this is completely justifiable but the rationale needs to be addressed more directly in discussing the methods- see suggested approach in the detailed comments.

Table 4. See the comments in the attached review on Table 4- it appears that the authors have yet to complete this table- the large number of empty cells suggests this is a work in progress. For a paper comparing experience and lessons learned across three country projects, having no information/observation/lesson learned for one of the countries for every one of the topics ( the key factors in scale up and sustainability put forth by the paper) in the table -except feasibility- is problematic. This is very surprising and would suggest a possible lack of depth in the analysis if this is indeed the case. In effect the key conclusions would be based on only two of the three cases. If there is no information the empty cells should be labelled as such e.g. N.A.

**Minor essential revisions**

Line 457 repeats line 410 on previous page—the section titles are the same – one needs to be changed at least slightly.

**Discretionary revisions**

See the attached review for comments and suggestions concerning potential revisions to strengthen the paper.

**Additional comments**

The manuscript addresses an extremely timely and important issue in global health today- that of how to best strengthen the planning for and the process of scale up of health interventions/innovations in the context of weak health systems- and to ensure that the gains achieved are sustained. There has been an emerging literature on this topic over the last decade and there is a need for more comparative country case studies.

The manuscript is extremely well written and easy to read- a pleasure, which in some places tends to hide some important areas in need of revision or strengthening in the paper. I highlight some of these as follows:

The title and abstract promise the reader important insights on scaling up and sustainability, but much to this readers dismay, the paper provides very little description of what if anything was actually scaled up as opposed to what was tested in the IR and considered for scaling up in each of the countries. Line 59 in the Abstract suggests that strategies were developed and scaling up took place as does line 178 in the text. However, neither the text, nor that of Table 2 provides adequate information to the reader to allow them understand about the interventions in sufficient detail, to understand how broadly they were initially implemented, to know what the final model was that was scaled up, to understand how the scaling up strategy was developed and what it involved in sufficient detail, to know anything significant about the process of scaling up or to appreciate the lessons learned through scaling. Although it is implied in various places in describing the projects, it is not clear that any of the project interventions were actually scaled up – although they do state that the intervention in Afghanistan was further tested by the government in other cultural/linguistic areas before making a decision on whether to expand the community health score card. Otherwise it appears that the paper is actually describing a process of pilot testing or IR to learn and determine what might work in the specific country contexts and what might be scaled up in the future, together with some of the other considerations such as inclusion of a wide range of key stakeholders. This it should be acknowledged more directly in the title and the ensuing text of the manuscript.

As for sustainability, the authors state that as the projects have just ended it is not possible to discuss sustainability more thoroughly, beyond stating that involving key stakeholders will likely to contribute to future sustainability. I think that if the title is to be retained than further description of the process of scale up and of sustainability is essential.

Alternatively if the three projects ended before the implications of the IR for scaling up and sustainability could be directly assessed (as stated clearly for the first time at the beginning of the discussion), then the title could/should reflect the paper’s major message which appears to be that implementation research (IR) can make a valuable contribution in the testing of interventions implemented on a small scale so as to ensure that adequate information concerning the interventions’ feasibility, effectiveness and acceptability to both target populations and the health system implementing the interventions (e.g. all of the relevant stakeholders) is obtained. This is a critical precursor to systematically planning a scaling up strategy that is be likely to be successful for both expansion and institutionalization. Both types of scale up are typically necessary (except in rare circumstances) for broad coverage and for sustainability.

The results section of the abstract emphasizes that the three cases differed and how an IR perspective led to a wider range of insights than merely focusing on feasibility and effectiveness, but should highlight that the finding that whether explicit or not learning about acceptability, cost effectiveness, feasibility in real life programs and sustainability need to be central focuses of IR to support future scale up.

*The Introduction* provides a very good short summary of the recent focus on scale up frameworks and the value of IR for strengthen health programs in weak heath systems. The paper states that it draws heavily on the framework presented in the Simmons et al. ExpandNet/WHO 2007 publication (reference 9)- but the citation in the reference list is missing. I believe it should be as follows:

(9) Simmons R, Fajans P, Ghiron L (Eds). Scaling up health service delivery: from pilot innovations to policies and programmes; Geneva: World Health Organization; 2007. 183 p.

Given this emphasis on the ExpandNet/WHO framework as background for the paper it is surprising that the authors do not refer to any of the later ExpandNet/WHO reference documents which provide practical guidance on scaling up and discuss in detail many of the issues raised or discussed later in the paper. For example see the following:

WHO/RHR/ExpandNet. *Practical guidance for scaling up health service innovations*. Geneva: World Health Organization; 2009, 62 p.

WHO/RHR/ExpandNet, World Health Organization. *Nine steps for developing a scaling-up strategy*. Geneva: World Health Organization; 2010. 35 p.

WHO/RHR/ExpandNet. *Beginning with the end in mind: planning pilot projects and other programmatic research for successful scaling up*. Geneva: World Health Organization; 2011. 18 p.

The last document (BWEIM) would seem to be a particularly important reference as it focuses on the design of pilot, demonstration and implementation research projects so as to increase the prospects for future sustainable scale up of the interventions being tested. It discusses 12 recommendations drawn from the literature and experience, which include those presented in the manuscript, but go well beyond in addition. For example, the first is to include a wide range of relevant stakeholders, including beneficiaries and implementers in the design and implementation of the pilot, demonstration or IR project. Another discusses the importance of gaining understanding of the socio-cultural, geographic and institutional contexts in which scaling up will be expected to take place.

Page 4 line 132-142 Clarify--Simmons et al. discuss four types of scaling up. An effective and sustainable scaling up strategy is likely to combine both horizontal and vertical types if sustainability is to be achieved. Over time during the process of scaling up diversification is often added. These are not mutually exclusive types of scaling up. At the same time spontaneous scaling up can be consider a type of scaling up but it represents a lack of a systematic strategy.

Page 6 line 178 here as elsewhere “informed the process of scaling up” suggests that scaling up actually took place in the three countries.

Page 6 line 200- you might want to consider deconstructing Appropriateness (also in Table 1) to explicitly include effectiveness, feasibility in the cultural and institutional context, relevant, acceptable to stakeholders-both target communities and providers.

*The Methods* of the study are well described. It would be important that the “rich descriptions” developed by the author be available on line to interested readers- the amount of description provided in Table 2 is in many cases not sufficient for the Reader to adequately understand the context, what was done in the projects- whether IR studies or in scaling up, particularly for drawing comparative lessons.

Some might argue that having the lead author who is the overall project head review the individual country reports and publications, and then develop the rich descriptions and lessons learned for review by the country teams would potentially lead to biases in interpretation of the experiences. This is acknowledged by the authors under study limitations, but no counter argument is given. I think that this should be further addressed either under methods or in the limitations section.

For example this could be done by more clearly discussing under the methods section that the paper represents a comparative case study based on participant observation and that the findings, conclusions and lessons learned represent those of the team implementing the projects. One could also refer to the fact that the recommendations emerging are consistent with the literature on scaling up and sustainability, supporting their potential validity.

*Results section*

The attributes of the interventions section describes very briefly the general nature of the interventions and how the focus of the project over the period of implementation- but not their attributes as referred to in Table 1 where attributes related to their appropriateness to the context and the actors and their fidelity to the original model as key research questions for IR are highlighted. Although the description of the evolution of the projects is certainly related to the need for adaptation, as opposed to fidelity of the interventions, this section does not adequately frame the observations in terms of fidelity/need for adaptation, which is a critical issue when discuss scaling up.

The authors may also wish to consider that when “attributes of the interventions” have typically been discussed in the literature on diffusion of innovation and later on concerning scalability in the literature on scaling up- this term most often refers to a set of characteristics including credibility, observability, relevance, relative advantage, ease of transfer, compatibility and testability.

The subsection describing the scaling up strategies does not provide adequate description to make for a useful discussion of major issues in strategy development, beyond noting that the projects did not have explicit strategies when they started. This is not surprising as each was testing a set of interventions for feasibility and effectiveness prior to what could be expected to be a systematic process of strategy development. Table 2 provides slightly more detail, but the lack of information about how the strategies were developed, who was involved, and how the strategies addressed key issues of expansion and institutionalization, the building of capacity of those supporting the process of scale up and of the implementation organizations, the intervention costs and sources of financing, M and E of the process of scup etc. etc. result in only a very superficial understanding of the strategies and their potential for sustainability.

Again it is not clear if these strategies were actually implemented to a significant degree.

Actors- this section together with Table 3, describes in considerable detail how each of the three projects learned how important it was to identify and understand the complex array of stakeholders that needed to be involved in the IR and the subsequent scale up if it was to be successful and sustainable. Although this is a central point perhaps the section could be shortened somewhat with less specific detail, as this point is so well documented across the scale up literature, and more detailed description is needed elsewhere in the paper.

Contribution of IR

See comments later on Table 4

P. 15 line 448- 453--The argument that the original objectives of the IR projects- feasibility, effectiveness and acceptability were too narrow but because of the researcher’s long standing association with the country contexts they learned a lot of critical information which resulted in actionable lessons is very important. It does not explain why they didn’t design more effective, acceptable projects in the first place. What led them to gain the new insights during the course of the projects. This highlights the importance of tools such as the WHO/ExpandNet Beginning With the End In Mind document with its recommendations and its checklist to help ensure that those designing the IR take into account critical issues that influence later scale up and sustainability.

*Discussion*

Line 457 repeats line 410 on previous page—same section title – one needs to be changed.

Line 478 to 483 The complexity of scaling up health interventions in complex systems is a strong argument for the need for concrete systematic approaches to the development of specific scaling up strategies adapted to these systems. See the WHO/ExpandNet document *Nine Steps for developing a scaling up strategy* 2010, and others for discussion of the need for and the outline of such a process.

See also the publication Keyonzo et al “From Project to Program: Tupange’s Experience with Scaling Up Family Planning Interventions in Urban Kenya,” March 2015, Reproductive Health Matters for a case study of the benefits of a systematic approach to strategy development.

Im*plications for future research and practice*

Line 532-540- consider adding a number of other references as these lessons from the paper reflect lessons highlight in so many other previous publications not just in health systems research but also the scaling up research- see ref 9, WHO/ExpandNet Practical Guidance and BWEIM doc among others.

Line 547-571- the statement in line 547-549 (as well as the third sentence of the conclusion) represent a misinterpretation of the ExpandNet/WHO framework. The figure of the framework shows a resource team, which often initially led the pilot or IR research and now supports the scaling up process, and an implementing organization that is responsible for implementing the interventions on a wider scale. Although these are shown in the figure as separate conceptual entities for simplicity, the texts of the various WHO/ExpandNet documents make it clear that there needs to be overlap in their composition or membership. Members of the implementing organization need to work hand in had with the research team in the design and implementation of the pilot/demonstration/IR project and as scaling up proceeds, members of the implementing organization need to assume an ever increase role as resource team members supporting the scaling up process. This blending or blurring of teams that the authors note in their IR studies is documented good practice for promoting successful scale up.

This does lead to a very important point made by the authors in lines 559-571. Given the critical importance of participatory process in promoting scale up and sustainability; research relationships. research neutrality and research rigor need to be reconsidered if scale up is the objective. I find it useful to return to a point made in the paper by Madon et al (ref 13 in the manuscript) where they distinguish between the need for “proof of concept” studies and “proof of implementation” studies testing whether implementation of interventions is feasible, acceptable and effective under routine program conditions and with little in the way of external resources. In real life this is not bipolar, but rather a continuum of differences in attributes (see Keyonzo et al mentioned ealier), but following a successful pilot/demonstration/IR project showing a proof of programmatic concept, a less resourced (and less rigorous, but very much more participatory) “proof of implementation” project may well be required as the next step in moving forward with scale up.

*Conclusion*

The conclusion seems repetitive of points made/sentences earlier in the discussion. Consider strengthening the conclusion.

Table 1 The choice of research questions for the four dimensions seem quite arbitrary- how were these chosen? For example attributes of the innovation or service- appropriateness is important is certainly critical- Maintaining fidelity is an attribute of the scaling up process which can be important consider but the literature on scaling up including that cited by the authors suggests that adaptation is more likely to be critical for acceptability, effectiveness and sustainability.

Under attributes of the target community and other actors- coverage is really more of an attribute of the service, while socioeconomic and cultural contexts of communities and of the health institutional settings would seem to be central based both on the literature and the manuscript. In terms of scaling up strategy. implementation costs and financing are very important and this is critical for sustainability, but just as important are issues related to how to institutionalize the interventions in policies, budgets, programs, and operational guidelines. Addressing both expansion and institutionalization are now recognized as key aspects of scaling up.

Table 3- it is not clear that this table is necessary or adds substantially to the paper. The fact that it is important to include key actors from the community level, the district and national levels and the private sector in implementing IR or demonstration projects- and that these actors are different contexts -is the key message which can be communicated in text- who they were in each specific country does not seem to greatly contribute to the paper.

Table 4. Has this table been completed or is it a work in progress? It is not clear why there are so many empty cells here – there is only one key factor- feasibility- where all three countries have information or a lesson learned. If there were really no observations or lessons to be learned in so many cases then perhaps the information is better handled in the text of the manuscript. For a paper that intends to compare experience and lessons learned these is very little discussion of common threads or differences that emerge from this table. The discussion in the text tends to describe the project findings but not provide a comparative analysis of many of these other topics.

If the table is indeed complete and the empty cells do not have information they should be labeled as such e.g. N.A.

The content of the table is highly project specific and doesn’t draw out comparisons or lessons across the two or three projects for the factor in question.

**Declaration of competing interests**

I have no financial or other non-financial competing interests in reviewing this manuscript.

As a member of the ExpandNet secretariat I do have an interest in assuring that the work and materials developed by ExpandNet in collaboration with WHO/RHR is accurately referred to and represented in the text.

**Author’s response to reviews**

**Reviewer 1:** Kara Hanson

1. I was not convinced that these two different conceptual frameworks (attributes of / factors influencing scale-up and IR questions) are really “integrated” as claimed in the background section of the abstract, or whether they are really two different frameworks, applied separately to “work through” each of the three interventions looking to identify similarities and differences. (indeed, the tables presenting the results on scale up and IR are kept separate). It would be good to have further clarification on how this analysis really integrates these frameworks – and have this a more prominent part of the discussion section, so that the original contribution of the paper is more clear

***Response:***

Thank you for this thoughtful comment which made us return to the manuscript with a more critical eye. Indeed you are right that the tables were previously presented as two separate tables – with Table 2 presenting scaling up dimensions and Table 4 presenting IR findings. However, there were clearly links between Table 2 and Table 4, and in order to better integrate these two different conceptual frameworks we have now merged them in a series of country specific tables (Tables 2-4).

We have also added text to the discussion that more clearly articulates this contribution, lines 494-497

2. I did not feel that the analysis addressed how variations in scale-up attributes had informed/influenced different IR strategies, and I would like to see a bit more analysis of this, so as to come to a clearer understanding of the second point in objective (ii) – ie. how scale-up strategies could/should/would influence IR strategies.

***Response:***

This is another good point. Based upon such a small group of cases it is difficult to draw hard and fast conclusions on this issue, but we have added a short discussion about this point (lines 565-578) and also suggested that this is an area where further work should be done.

**Reviewer 2:** Peter Fajans

1. The manuscript addresses an extremely timely and important issues in global health today- that of how to best to strengthen the planning for and the process of scale up of health interventions/innovations in the context of weak health systems- and how to ensure that the gains achieved are sustained. There has been an emerging literature on this topic over the last decade and the recent attention to implementation research provides an opportunity to strengthen the design and implementation of pilot/demonstration and other programmatic research to test health interventions on a limited scale, but at the same time to provide the necessary learning to support future successful scale up and long term sustainability. There is also a need for more comparative country case studies of this process.

The manuscript is extremely well written and easy to read- a pleasure, which in some places tends to hide some significant shortcomings or areas of need in strengthening in the paper. These are discussed in detail in the attached review comments.

***Response:***

Thank you for your positive and careful review and the more detailed comments provided in the attachment

2. The primary compulsory revision is that there is a need for greater clarity in what the paper is actually discussing. The title and particularly the text in the first half of the manuscript seem to suggest that the paper is comparing the process of scaling up and sustainability of the interventions. Only when the reader reaches the discussion section does it seem to become clear that in fact the three projects appear to have ended prior to any systematic scaling up of the interventions, much less assessment of the actual sustainability of the interventions- whether on a initial small scale or following scaling up. Without this clarification the reader interested in scaling finds the lack of information about the development of the scaling up strategy or in depth discussion of factors related to sustainability to be problematic.

***Response:***

It is fair to say that in the previous version of the paper, it is only in the discussion that it is explicitly stated that the interventions described have not all been scaled up. In the revised text we have been much clearer about the status of scale up of the interventions and when first describing the interventions (lines 274-324) we have provided an up-to-date snapshot of the nature of scale up that has occurred. We also looked carefully at the language used throughout the text, but really did not think that this was misleading in the sense that we were talking about evidence relevant to scale up, if not actually evidence used directly in the scale up process. The reviewer is right about the lack of focus on sustainability, and we also note that sustainability is one of the IR questions embedded within scale up, so we have chosen to remove the term sustainability from the title.

3. The authors should more directly (but briefly) discuss the potential bias resulting from the lead author and the key country researchers involved in the projects reviewing the project reports, synthesizing rich descriptions which then serve as data for the comparative analysis. I think this is completely justifiable but the rationale needs to be addressed more directly in discussing the methods- see suggested approach in the detailed comments.

***Response:***

We have expanded the discussion of this limitation, lines 585-592.

4. Table 4. See the comments in the attached review on Table 4- it appears that the authors have yet to complete this table- the large number of empty cells suggests this is a work in progress. For a paper comparing experience and lessons learned across three country projects, having no information/observation/lesson learned for one of the countries for every one of the topics ( the key factors in scale up and sustainability put forth by the paper) in the table -except feasibility- is problematic. This is very surprising and would suggest a possible lack of depth in the analysis if this is indeed the case. In effect the key conclusions would be based on only two of the three cases. If there is no information the empty cells should be labelled as such e.g. N.A.

***Response:*** We believe that perhaps the reviewer overlooked one of the pages in the old Table 4. All of the country cases have data on at least four of the IR dimensions presented. Hopefully the new shorter, country-specific tables will be easier to navigate. We have also inserted text stating “not assessed” to make it clearer which dimensions were not assessed.

5. Line 457 repeats line 410 on previous page—the section titles are the same – one needs to be changed at least slightly.

***Response:*** Thank you – we have adapted one of the titles to avoid overlap.

6. See the attached review for comments and suggestions concerning potential revisions to strengthen the paper.

***Response:*** Thank you – please see our brief responses in the text below.

The manuscript addresses an extremely timely and important issue in global health today- that of how to best strengthen the planning for and the process of scale up of health interventions/innovations in the context of weak health systems- and to ensure that the gains achieved are sustained. There has been an emerging literature on this topic over the last decade and there is a need for more comparative country case studies.

The manuscript is extremely well written and easy to read- a pleasure, which in some places tends to hide some important areas in need of revision or strengthening in the paper. I highlight some of these as follows:

The title and abstract promise the reader important insights on scaling up and sustainability, but much to this readers dismay, the paper provides very little description of what if anything was actually scaled up as opposed to what was tested in the IR and considered for scaling up in each of the countries. Line 59 in the Abstract suggests that strategies were developed and scaling up took place as does line 178 in the text. However, neither the text, nor that of Table 2 provides adequate information to the reader to allow them understand about the interventions in sufficient detail, to understand how broadly they were initially implemented, to know what the final model was that was scaled up, to understand how the scaling up strategy was developed and what it involved in sufficient detail, to know anything significant about the process of scaling up or to appreciate the lessons learned through scaling. Although it is implied in various places in describing the projects, it is not clear that any of the project interventions were actually scaled up – although they do state that the intervention in Afghanistan was further tested by the government in other cultural/linguistic areas before making a decision on whether to expand the community health score card. Otherwise it appears that the paper is actually describing a process of pilot testing or IR to learn and determine what might work in the specific country contexts and what might be scaled up in the future, together with some of the other considerations such as inclusion of a wide range of key stakeholders. This it should be acknowledged more directly in the title and the ensuing text of the manuscript.

As for sustainability, the authors state that as the projects have just ended it is not possible to discuss sustainability more thoroughly, beyond stating that involving key stakeholders will likely to contribute to future sustainability. I think that if the title is to be retained than further description of the process of scale up and of sustainability is essential.

Alternatively if the three projects ended before the implications of the IR for scaling up and sustainability could be directly assessed (as stated clearly for the first time at the beginning of the discussion), then the title could/should reflect the paper’s major message which appears to be that implementation research (IR) can make a valuable contribution in the testing of interventions implemented on a small scale so as to ensure that adequate information concerning the interventions’ feasibility, effectiveness and acceptability to both target populations and the health system implementing the interventions (e.g. all of the relevant stakeholders) is obtained. This is a critical precursor to systematically planning a scaling up strategy that is be likely to be successful for both expansion and institutionalization. Both types of scale up are typically necessary (except in rare circumstances) for broad coverage and for sustainability.

**Response:** the points down to here were in the main form received from the reviewer and are addressed in the text above.

The results section of the abstract emphasizes that the three cases differed and how an IR perspective led to a wider range of insights than merely focusing on feasibility and effectiveness, but should highlight that the finding that whether explicit or not learning about acceptability, cost effectiveness, feasibility in real life programs and sustainability need to be central focuses of IR to support future scale up.

**Response:** we think this is implicit in the abstract, and we did not have sufficient words to make the point more explicitly

*The Introduction* provides a very good short summary of the recent focus on scale up frameworks and the value of IR for strengthen health programs in weak heath systems. The paper states that it draws heavily on the framework presented in the Simmons et al. ExpandNet/WHO 2007 publication (reference 9)- but the citation in the reference list is missing. I believe it should be as follows:

(9) Simmons R, Fajans P, Ghiron L (Eds). Scaling up health service delivery: from pilot innovations to policies and programmes; Geneva: World Health Organization; 2007. 183 p.

**Response:** Thank you we have added this in full.

Given this emphasis on the ExpandNet/WHO framework as background for the paper it is surprising that the authors do not refer to any of the later ExpandNet/WHO reference documents which provide practical guidance on scaling up and discuss in detail many of the issues raised or discussed later in the paper. For example see the following:

WHO/RHR/ExpandNet. *Practical guidance for scaling up health service innovations*. Geneva: World Health Organization; 2009, 62 p.

WHO/RHR/ExpandNet, World Health Organization. *Nine steps for developing a scaling-up strategy*. Geneva: World Health Organization; 2010. 35 p.

WHO/RHR/ExpandNet. *Beginning with the end in mind: planning pilot projects and other programmatic research for successful scaling up*. Geneva: World Health Organization; 2011. 18 p.

**Response:** While we appreciate that there is a broader array of ExpandNet publications, we have already referenced the one found most useful

The last document (BWEIM) would seem to be a particularly important reference as it focuses on the design of pilot, demonstration and implementation research projects so as to increase the prospects for future sustainable scale up of the interventions being tested. It discusses 12 recommendations drawn from the literature and experience, which include those presented in the manuscript, but go well beyond in addition. For example, the first is to include a wide range of relevant stakeholders, including beneficiaries and implementers in the design and implementation of the pilot, demonstration or IR project. Another discusses the importance of gaining understanding of the socio-cultural, geographic and institutional contexts in which scaling up will be expected to take place.

Page 4 line 132-142 Clarify--Simmons et al. discuss four types of scaling up. An effective and sustainable scaling up strategy is likely to combine both horizontal and vertical types if sustainability is to be achieved. Over time during the process of scaling up diversification is often added. These are not mutually exclusive types of scaling up. At the same time spontaneous scaling up can be consider a type of scaling up but it represents a lack of a systematic strategy.

**Response:** thank you, we have amended the text lines 143-146 to make this clear

Page 6 line 178 here as elsewhere “informed the process of scaling up” suggests that scaling up actually took place in the three countries.

**Response:** addressed above

Page 6 line 200- you might want to consider deconstructing Appropriateness (also in Table 1) to explicitly include effectiveness, feasibility in the cultural and institutional context, relevant, acceptable to stakeholders-both target communities and providers.

**Response:** Table 1 is derived from another publication (Peters et al). We had previously forgotten to add the source to the table. We have now done this….but do not think it is appropriate to adjust the categories used in Peters et al.

*The Methods* of the study are well described. It would be important that the “rich descriptions” developed by the author be available on line to interested readers- the amount of description provided in Table 2 is in many cases not sufficient for the Reader to adequately understand the context, what was done in the projects- whether IR studies or in scaling up, particularly for drawing comparative lessons.

**Response:** Done – added as online annexes.

Some might argue that having the lead author who is the overall project head review the individual country reports and publications, and then develop the rich descriptions and lessons learned for review by the country teams would potentially lead to biases in interpretation of the experiences. This is acknowledged by the authors under study limitations, but no counter argument is given. I think that this should be further addressed either under methods or in the limitations section.

For example this could be done by more clearly discussing under the methods section that the paper represents a comparative case study based on participant observation and that the findings, conclusions and lessons learned represent those of the team implementing the projects. One could also refer to the fact that the recommendations emerging are consistent with the literature on scaling up and sustainability, supporting their potential validity.

**Response:** addressed above

*Results section*

The attributes of the interventions section describes very briefly the general nature of the interventions and how the focus of the project over the period of implementation- but not their attributes as referred to in Table 1 where attributes related to their appropriateness to the context and the actors and their fidelity to the original model as key research questions for IR are highlighted. Although the description of the evolution of the projects is certainly related to the need for adaptation, as opposed to fidelity of the interventions, this section does not adequately frame the observations in terms of fidelity/need for adaptation, which is a critical issue when discuss scaling up.

**Response:** we felt this point was better made in the discussion section and have added it there lines 523-529

The authors may also wish to consider that when “attributes of the interventions” have typically been discussed in the literature on diffusion of innovation and later on concerning scalability in the literature on scaling up- this term most often refers to a set of characteristics including credibility, observability, relevance, relative advantage, ease of transfer, compatibility and testability.

**Response:** we have decided that this was not critical to the text, and would add unnecessary words.

The subsection describing the scaling up strategies does not provide adequate description to make for a useful discussion of major issues in strategy development, beyond noting that the projects did not have explicit strategies when they started. This is not surprising as each was testing a set of interventions for feasibility and effectiveness prior to what could be expected to be a systematic process of strategy development. Table 2 provides slightly more detail, but the lack of information about how the strategies were developed, who was involved, and how the strategies addressed key issues of expansion and institutionalization, the building of capacity of those supporting the process of scale up and of the implementation organizations, the intervention costs and sources of financing, M and E of the process of scup etc. etc. result in only a very superficial understanding of the strategies and their potential for sustainability.

Again it is not clear if these strategies were actually implemented to a significant degree.

**Response:** Addressed above

Actors- this section together with Table 3, describes in considerable detail how each of the three projects learned how important it was to identify and understand the complex array of stakeholders that needed to be involved in the IR and the subsequent scale up if it was to be successful and sustainable. Although this is a central point perhaps the section could be shortened somewhat with less specific detail, as this point is so well documented across the scale up literature, and more detailed description is needed elsewhere in the paper.

**Response:** we think there is some value in this table and have retained it

Contribution of IR

See comments later on Table 4

P. 15 line 448- 453--The argument that the original objectives of the IR projects- feasibility, effectiveness and acceptability were too narrow but because of the researcher’s long standing association with the country contexts they learned a lot of critical information which resulted in actionable lessons is very important. It does not explain why they didn’t design more effective, acceptable projects in the first place. What led them to gain the new insights during the course of the projects. This highlights the importance of tools such as the WHO/ExpandNet Beginning With the End In Mind document with its recommendations and its checklist to help ensure that those designing the IR take into account critical issues that influence later scale up and sustainability.

*Discussion*

Line 457 repeats line 410 on previous page—same section title – one needs to be changed.

**Response:** addressed above

Line 478 to 483 The complexity of scaling up health interventions in complex systems is a strong argument for the need for concrete systematic approaches to the development of specific scaling up strategies adapted to these systems. See the WHO/ExpandNet document *Nine Steps for developing a scaling up strategy* 2010, and others for discussion of the need for and the outline of such a process.

See also the publication Keyonzo et al “From Project to Program: Tupange’s Experience with Scaling Up Family Planning Interventions in Urban Kenya,” March 2015, Reproductive Health Matters for a case study of the benefits of a systematic approach to strategy development.

Im*plications for future research and practice*

Line 532-540- consider adding a number of other references as these lessons from the paper reflect lessons highlight in so many other previous publications not just in health systems research but also the scaling up research- see ref 9, WHO/ExpandNet Practical Guidance and BWEIM doc among others.

Line 547-571- the statement in line 547-549 (as well as the third sentence of the conclusion) represent a misinterpretation of the ExpandNet/WHO framework. The figure of the framework shows a resource team, which often initially led the pilot or IR research and now supports the scaling up process, and an implementing organization that is responsible for implementing the interventions on a wider scale. Although these are shown in the figure as separate conceptual entities for simplicity, the texts of the various WHO/ExpandNet documents make it clear that there needs to be overlap in their composition or membership. Members of the implementing organization need to work hand in had with the research team in the design and implementation of the pilot/demonstration/IR project and as scaling up proceeds, members of the implementing organization need to assume an ever increase role as resource team members supporting the scaling up process. This blending or blurring of teams that the authors note in their IR studies is documented good practice for promoting successful scale up.

**Response:** We went back to the Expandnet publications and actually they do make a very clear distinction between the resource team and the user organization so we have elected to keep the language as it is.

This does lead to a very important point made by the authors in lines 559-571. Given the critical importance of participatory process in promoting scale up and sustainability; research relationships. research neutrality and research rigor need to be reconsidered if scale up is the objective. I find it useful to return to a point made in the paper by Madon et al (ref 13 in the manuscript) where they distinguish between the need for “proof of concept” studies and “proof of implementation” studies testing whether implementation of interventions is feasible, acceptable and effective under routine program conditions and with little in the way of external resources. In real life this is not bipolar, but rather a continuum of differences in attributes (see Keyonzo et al mentioned ealier), but following a successful pilot/demonstration/IR project showing a proof of programmatic concept, a less resourced (and less rigorous, but very much more participatory) “proof of implementation” project may well be required as the next step in moving forward with scale up.

*Conclusion*

The conclusion seems repetitive of points made/sentences earlier in the discussion. Consider strengthening the conclusion.

Table 1 The choice of research questions for the four dimensions seem quite arbitrary- how were these chosen? For example attributes of the innovation or service- appropriateness is important is certainly critical- Maintaining fidelity is an attribute of the scaling up process which can be important consider but the literature on scaling up including that cited by the authors suggests that adaptation is more likely to be critical for acceptability, effectiveness and sustainability.

**Response:** Hopefully the addition of the source to this table will make it clearer.

Under attributes of the target community and other actors- coverage is really more of an attribute of the service, while socioeconomic and cultural contexts of communities and of the health institutional settings would seem to be central based both on the literature and the manuscript. In terms of scaling up strategy. implementation costs and financing are very important and this is critical for sustainability, but just as important are issues related to how to institutionalize the interventions in policies, budgets, programs, and operational guidelines. Addressing both expansion and institutionalization are now recognized as key aspects of scaling up.

Table 3- it is not clear that this table is necessary or adds substantially to the paper. The fact that it is important to include key actors from the community level, the district and national levels and the private sector in implementing IR or demonstration projects- and that these actors are different contexts -is the key message which can be communicated in text- who they were in each specific country does not seem to greatly contribute to the paper.

**Response:** Addressed above

Table 4. Has this table been completed or is it a work in progress? It is not clear why there are so many empty cells here – there is only one key factor- feasibility- where all three countries have information or a lesson learned. If there were really no observations or lessons to be learned in so many cases then perhaps the information is better handled in the text of the manuscript. For a paper that intends to compare experience and lessons learned these is very little discussion of common threads or differences that emerge from this table. The discussion in the text tends to describe the project findings but not provide a comparative analysis of many of these other topics.

If the table is indeed complete and the empty cells do not have information they should be labeled as such e.g. N.A.

**Response:** Addressed above

The content of the table is highly project specific and doesn’t draw out comparisons or lessons across the two or three projects for the factor in question.
